# Clinical and radiographic outcomes of non-surgical retreatment of mature maxillary incisors using two regenerative endodontic techniques in adolescents: a 24-month randomized clinical trial

**DOI:** 10.1038/s41405-025-00324-w

**Published:** 2025-04-11

**Authors:** Ahmad Abdel Hamid Elheeny, Sherif Shafik EL Bahnasy, Yassmin Mohamed ElMakawi, Mohammed Turky, Eman Farouk Ahmed, Norhan Khaled Omar Wahba

**Affiliations:** 1 Assistant Professor of Paediatric and Community Dentistry, Faculty of Dentistry, Minia University, Minia, Egypt; 2 Assistant Professor of Paediatric and Community Dentistry, Faculty of Dentistry, Sphinx University, Sphinx, Egypt; 3 Assistant Professor of Oral Radiology, Faculty of dentistry, The British university in Egypt, Al Shorouk City, Egypt; 4 Lecturer of Paediatric and Community Dentistry, Faculty of Oral and Dental Medicine, Nahda University, Nahda, Egypt; 5 Department of Endodontics, Faculty of Dentistry, Minia University, Minia, Egypt; 6 Assistant Professor of Microbiology and Immunology, Microbiology and Immunology Department, Faculty of Pharmacy, Sohag University, 82524 Province, Sohag Egypt; 7 Demonstrator of Paediatric and Community Dentistry, Faculty of Oral and Dental Medicine, Nahda University, Nahda, Egypt

**Keywords:** Paediatric dentistry, Dentistry

## Abstract

**Aims:**

The primary aim was to monitor the healing of the periapical radiolucencies of adolescents’ mature permanent teeth with apical periodontitis after root canal retreatment with two REPs techniques at 24 months of follow-up. The secondary aim was to assess clinical outcomes and positive responses of retreated teeth to pulp sensibility tests.

**Methodology:**

Forty adolescents with 48 teeth were enroled and randomly allocated into two equal groups after being matched according to their periapical index (PAI) scores. Root canal retreatment was performed with blood clot (BC) formation in one group and platelet-rich fibrin (PRF) in the other group. The healing process was tracked using standardized two-dimensional radiographic images to record the changes in the PAI scores after 3, 6, 12, and 24 months. Additionally, the clinical signs and symptoms and the positive responses to pulp sensibility tests were monitored. The difference between the PAI medians was analysed using the Mann–Whitney U test. The main impact of time on the PAI values and the interaction between time and the REPs technique were assessed using the general linear model (GLM). The alpha level of significance was 5%.

**Results:**

After two years of follow-up, there was no significant difference between the two groups clinically and in the PAI medians. The overall success rates in the BC and PRF groups were 95% and 100%, respectively (*P* > 0.05). Positive pulp responses were detected in 71% of the BC group and 73% in the PRF group (*P* > 0.05). The EPT mean values in the BC and PRF groups were 40.86 ± 6.60 and 37.9 ± 15.22, respectively (*P* > 0.05). Time had a significant impact on the PAI scores over the follow-up periods (*P* > 0.0001), while the interaction effect of time with the REPs technique had no significant effect on the PAI scores (*P* = 0.126).

**Conclusions:**

REPs were effective in the retreatment of mature maxillary permanent incisors with apical periodontitis with a comparable reduction in the periapical radiolucencies and clinical outcomes associated with approximately similar positive responses to thermal and electric pulp tests.

## Introduction

The emergence of tissue engineering in medicine has created new opportunities for its application in regenerative endodontic procedures (REPs) for immature permanent teeth, particularly those affected by pulp-periapical diseases. REPs rely on the biological integration of the three fundamental components of the tissue engineering triad: stem cells, biomimetic scaffolds, and bioactive growth factors [1]. This interplay initiates the repair process and facilitates the regeneration of neurovascular pulp-like tissues with immunological and proprioceptive functions [2].

Provocation of blood from the periapical region and allowing blood clot (BC) formation into a disinfectant root canal space is the regular strategy for revascularization [2]. The BC scaffold serves as a three-dimensional biological structure that facilitates and reinforces the migration, proliferation, and differentiation of stem cells [3, 4]. Furthermore, BC is the source of growth factors essential for regulating cellular function [1]. Nonetheless, attaining a sufficient amount of intracanal bleeding is not always feasible and is influenced by the severity of apical periodontitis [5]. Another limitation of the intracanal bleeding induction technique is the release of intracellular enzymes during apoptosis of hematopoietic cells, which may negatively impact stem cell survival [6].

To overcome these challenges, first, second, and third generations of autologous platelet-driven scaffolds, platelet-rich plasma (PRP), platelet-rich fibrin (PRF), and concentrated growth factor (CGF) have been introduced [7]. These platelet concentrates provide a three-dimensional complex fibrin mesh with slow biodegradation [8]. The fibrin meshwork architecture supports the migration of stem cells and allows the capturing of leukocytes and cytokines with small number of lymphocytes that help regulate inflammatory and infectious processes [9]. Moreover, this structure of PRF plays a crucial role in increasing the levels of growth factor release in a sustained manner, which synchronizes with the cellular ingrowth [10]. However, the clinical use of PRF may be hampered by the need for enduring venepuncture to collect the venous blood and the low mechanical properties to support the coronal restoration [6].

While REPs were initially developed for immature teeth with open apices, the principles underlying these procedures can be extrapolated to mature teeth with apical periodontitis. For instance, REPs could potentially stimulate the regeneration of damaged periapical tissues and even partially revitalize the pulp in mature teeth. The minimally invasive disinfection approach, combined with calcium hydroxide placement as an intracanal medicament, is equally applicable to both immature and mature permanent teeth [11]. Both types of teeth benefit from inflammation resolution and the promotion of periapical healing. REPs have demonstrated success in achieving apical closure and periapical healing in immature teeth, and similar success and survival rates are being explored in mature teeth [12].

The favourable outcomes of REPs in the treatment of immature permanent teeth have recently encouraged applying this approach in treating mature permanent teeth. Previous clinical trials that used REPs in the treatment of mature permanent teeth showed remarkable outcomes with high clinical and radiographic success rates. For example, Youssef et al. [5] reported the diminishing size of periapical lesions of 20 necrotic mature permanent incisors after treatment with BC and PRF scaffolds over one year of follow-up. Another trial showed a clinical and radiographic success rate of 92% in twenty-six mature permanent incisors with preoperative apical periodontitis compared to 80% in teeth treated with conventional root canal therapy [13]. Lu et al. [14], who retrospectively evaluated the long-term outcomes of REPs of 37 mature permanent teeth with an average of four years, reported a success rate of 89.2% with a significant reduction in the periapical index (PAI) values.

There is a shortage of data addressing the use of REPs in the retreatment of mature teeth. According to the available data, only one randomized clinical trial compared the clinical outcomes of mature maxillary permanent incisors, showing clinical success rates of 93.9% and 97% in teeth retreated with BC formation and conventional secondary root canal treatment, respectively [15]. To harness the advantages of REPs and provide a new treatment modality for managing non-vital permanent teeth with apical periodontitis, the primary aim of the current randomized clinical trial was to monitor the healing of periapical radiolucencies in adolescents’ mature permanent teeth with apical periodontitis after root canal retreatment with two REPs techniques over 24 months of follow-up. The secondary aim was to assess clinical outcomes and positive responses of retreated teeth to pulp sensibility tests [15].

To harness the advantages of REPs and provide a new treatment modality for managing non-vital permanent teeth with apical periodontitis, the primary aim of the current randomized clinical trial was to monitor the healing of periapical radiolucencies in adolescents’ mature permanent teeth with apical periodontitis after root canal retreatment with two REPs techniques over 24 months of follow-up. The secondary aim was to assess clinical outcomes and positive responses of retreated teeth to pulp sensibility tests.

## Materials and methods

### Ethical aspects

**Fig. 1:**
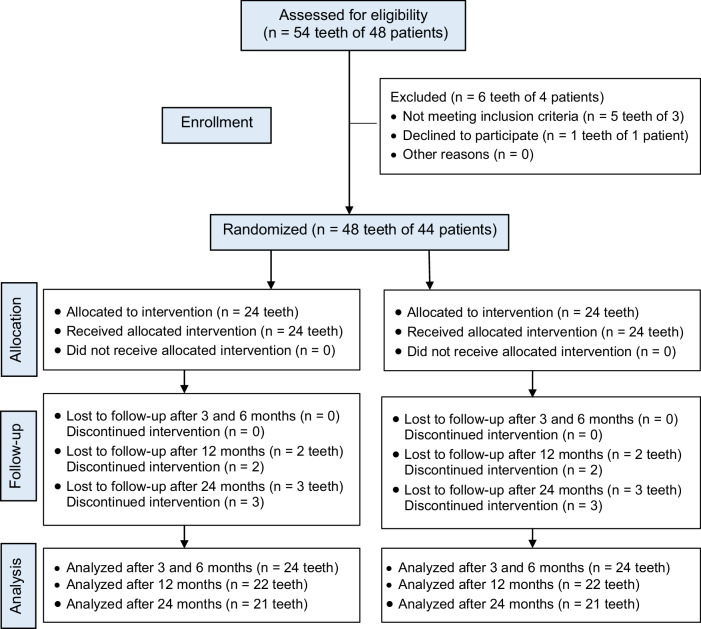
CONSORT flow chart of the randomized clinical trial.

In compliance with CONSORT 2010 guideline, the manuscript was written as illustrated in Fig. 1. The ethics committee of the local institution study reviewed and accredited the current clinical trial (Ethics Committee of the Faculty of Dentistry, Minia University, reference number 778). The trial commenced on July 26, 2022, and completed on January 8, 2024. Before inclusion in the study, each participant’s parent or legal guardian provided informed consent after receiving a thorough explanation of the study’s purpose, procedures, benefits, potential risks, and alternative treatment options in case of failure.

### Sampling, randomization, and allocation

The mean difference in the reduction of periapical radiolucencies using the PAI scores between sixteen teeth with apical periodontitis from 13 patients that have been retreated with BC or PRF, respectively, was 0.51 mm, with a pooled standard deviation (SD) of 0.89 mm. A 20 percent was added since more than one tooth per patient was included (i.e., an intraclass effect). An additional 20 percent was added to compensate for potential drop-off. Using G*Power 3.1.9.7 software, at an alpha level of significance of 5 percent and a power of 80 percent, a total of 48 maxillary central incisors (24 teeth per group) were included to be retreated with BC or PRF approaches.

According to preoperative maximum lesion diameters, teeth were matched using the PAI scores [16]. A computer-generated software “https://www.sealedenvelope.com” was used to perform permuted block randomization. An independent investigator who was obscured to study goals and clinical steps generated the sequence of randomization. The randomization procedures were the responsibility of an independent nurse who was concealed from the trial’s aim, protocol, and randomization sequence. To guarantee obtaining match groups based on the treatment group and the PAI scores, a block size of four was deemed. For that purpose, four double-folded sheets wrapped within identical opaque sealed envelopes that have been sequentially numbered. Each matched block consisted of four envelopes that were blended and transferred to four identical containers. The participant’s guardian randomly selected one envelope. Masking the treatment nature to the operator and participants was not available.

### Patient selection

#### Inclusion criteria

- Enroled adolescents aged 11 to less than 18 years old (class I according to the American Society of Anesthesiologists) with negative medical history of genetic, allergic, or systemic conditions.
- Single-rooted mature maxillary central incisors with apical foramen between 0.5 mm and less than 1.0 mm and root length ranged from 11$-$13 mm for standardization.
- Teeth with primary root canal treatment performed 1$-$2 years before failure were selected. The cause of failure was the fracture of the tooth structure adjacent to the coronal restoration or the loss or fracture of the coronal restoration.
- Teeth with asymptomatic apical periodontitis that was scored ≥3 according to the PAI.

#### Exclusion criteria

- Unrestorable crowns with a need for intracanal post placement were excluded.
- Positive history of traumatic dental injury, periodontal involvement, and extensive mobility (i.e., grade III mobility).
- Evidence of calcified root canals and the presence of pulp stones.
- Inability for total removal of the intracanal filling material or the presence of intracanal fractured instruments that couldn’t be retrieved.

## Clinical steps

According to the REPs proposed by the American Association of Endodontists (AAE), the clinical phases were carried out for both groups over the course of two visits [17].

### Root canal filling material removal

After the tooth was anesthetized with 4% articaine hydrochloride and 1:100,000 epinephrine (Septocaine®, SEPTODONT Ltd., Paris, France), a rubber dam was placed. In order to enable straight-line access to the canal orifice, the complete coronal restoration was then removed, and the access cavity was modified in accordance with the conventional standards of access cavity preparation [18]. An appropriately sized stainless-steel H-file (MANI Inc., Tochigi, Japan) was used for complete removal of the root canal filling material (no chemical solvents were employed) [19]. At this stage, a PA image was taken to confirm the complete root canal clearance and determine the working length with the aid of an electronic apex locator (DentaPort ZX, J. Morita Corp., Kyoto, Japan).

### Root canal disinfection regime

At the first appointment, the root canal was disinfected with alternating solutions of 20 mL of 1.5% NaOCl and 20 mL of 17% ethylenediaminetetraacetic acid (17% EDTA) (Prevest, DenPro, Jammu, India) for 5 minutes each, and 0.9% saline was used as a final irrigant to flush the root canal. Root canal was irrigated using a side-vented needle (gauge 30) (Zhucheng Binfei Medi-Tek Co., Ltd., Shandong, China). The needle was adjusted to stop at 1$-\,$ mm shorter than the working length. After drying the root canal with suitably-sized paper points (Meta Biomed, Korea), a non-settable calcium hydroxide (UltraCal, Ultradent, Utah, USA) intracanal dressing was packed into the root canal space to the predetermined working length. A light-cured resin glass ionomer restoration (GC Fuji II LC, Tokyo, Japan) applied and filled the access cavity down to the level of the cementoenamel junction. After 3 weeks the second appointment was rescheduled. The tooth was anesthetized with plain local anaesthesia (3% mepivacaine Scandonest®, Septodont, Saint-Maur-des-Fosses, France). Rubber dam was placed for isolation, and the root canal space was rinsed with 0.9% saline (5 mL) and 17% EDTA that was passively activated in 3 successive rounds (20 s each) with an ultrasonic non-cutting tip (E1 Irrisonic tip; Helse Dental Technology, São Paulo, SP, Brazil).

### REPs techniques

#### BC group

A sterile K-file #35 extended 2$-\,$ mm below the apical foramen, inducing bleeding from the periapical tissues. The bleeding filled the root canal space up to 3$-\,$ mm below the cementoenamel junction. The tip of the manual file was dipped in 17% EDTA and twisted slightly in case the amount of induced bleeding in the root canal space was insufficient.

#### PRF group

**Fig. 2:**
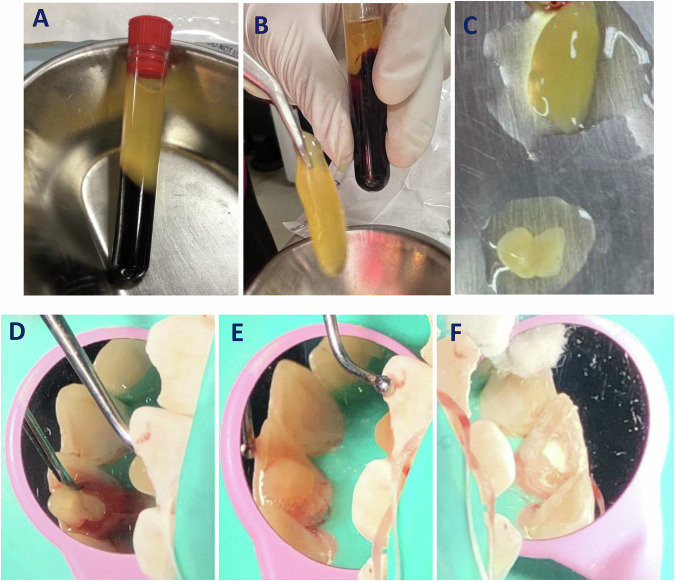
Preparation of platelet-rich fibrin clot and its placement in the root canal. **A** The three distinct blood layers of centrifuged blood samples at 3000 rpm for 10 min after being collected into sterile, plain-type, single-use centrifuge tubes. **B** Isolated yellow layer of PRF with an HTS 171c66.25 curved stainless steel tweezer. **C** The isolated PRF clot cut into smaller fragment. **D**, **E** PRF clot fragments were immediately introduced into the root canal using a small hand plugger. **F** a 3 mm layer thickness of Biodentine (Biodentine®, packed over the PRF.

A venous blood sample of 5 mL volume was drawn from the antecubital vein after venipuncture with a sterile hypodermic needle (Luer-Slip type, El Dawlia ico nos. 19 and 67). The blood samples were centrifuged (MSLZL09 MedGroup Guangzhou Medsinglong Medical Equipment, GuangZhou, China) at 3000 rpm for 10 min after being collected into sterile, plain-type, single-use centrifuge tubes (JIANGSU HXRT MD Co., Ltd. Taizhou, China). Of the three distinct blood layers, the middle fibrin-gelatinous yellow layer of PRF was isolated using an HTS 171c66.25 curved stainless steel tweezer (Fig. 2A, B). The isolated PRF clot was compressed using sterile gauze to eliminate excess fluids and cut into smaller fragments as required to be easily packed into the narrow root canal (Fig. 2C). PRF clot fragments were immediately introduced into the root canal using a small hand plugger (Dentsply Maillefer, Ballaigues, Switzerland) over freshly induced bleeding (i.e., blood clot was not formed) (Figure D and E) into the root canal space that has been elicited in a similar approach to BC formation after violating the root canal terminus with a K-file size 35. In case of failing to induce bleeding from the periapical region, the K-file was slightly bent after dipping in 17% EDTA [20].

For both groups, up to the level of the CEJ, a 3 mm layer thickness of Biodentine (Biodentine®, Septodont, Saint-Maur-des-Fossés, France) (Fig. 2F) was prepared according to manufacturer guidelines and placed over the BC or PRF with the aid of a collagen resorbable matrix (Collacone®, Botiss Biomaterials, Berlin, Germany), which served as a solid absorbable matrix. A base of light-cured resin-modified glass ionomer restorative material (Riva light cure, SDI, Bayswater, Australia) and composite resin restoration (Tetric N Ceram Bulk Fill, Ivoclar, Vivadent AG, Schaan, Liechtenstein) were used to seal the access cavity.

### Radiographic assessment using periapical radiographs (PAs)

For standardization issues, the radiographic parameters were adjusted for all participants to 60 kVp, 6 mA, 0.05 s exposure time, and a 0.4 mm focal spot size using the BelmontTM 2 X-ray machine. Additionally, a silicon-based radiographic stent was customized for each patient to guarantee a similar position of the digital phosphor plate (size 2) at every exposure. A parallel radiographic approach was considered via using a film holder that was attached to the digital plate. The Vista Scan system and software (DÜRR DENTALTM, Bietigheim-Bissingen, Germany) were used to process the intraoral PAs. The PAs were taken preoperatively and at 3, 6, 18, and 24 months to screen the healing progress.

### Thermal and electric pulp testing

Before performing TPT or EPT, the baseline records were defined, assessing the pulp sensibility of the contralateral intact tooth. Cotton rolls were placed in the mucobuccal fold for tooth isolation and then dried. At the middle third of the buccal surface, TPT and EPT were performed. For TPT, a cold stimulus using a small cotton pellet saturated with Green Endo-Ice refrigerant (Coltene/Whalkedent Inc., Cuyahoga Falls, Ohio, USA) for 5 s was performed. For EPT, the probe tip of the EPT (Parkell D640 Digitest II Pulp Vitality Tester, Brentwood, New York, USA) was dipped with a layer of toothpaste and the reading was obtained. The records of pulp sensibility tests were reported at each follow-up interval.

### Definition of the outcomes

At each follow-up period, each tooth was assessed radiographically for the reduction in the periapical radiolucency using PAI scores. Failed retreatment was defined on the basis of PAI value at each follow-up period that exceeded the baseline PAI score and was associated with positive evidence of clinical failure(s) [21]. The criteria of PAI according to Ørstavik et al. [16] were defined using the following scoring system: score “1” refers to normal periapical structure; score “2” indicates minor changes in the periapical bone structure; score “3” indicates bone changes with mineral bone loss; score “4” indicates apical periodontitis with well-defined radiolucency; and score “5” indicates apical periodontitis with diffuse exacerbating features.

Teeth with uncertain decreases in the radiographic periapical lesion dimensions or stable lesion dimensions with no positive evidence of clinical signs and symptoms were rescheduled for the next follow-up intervals. Failed teeth were referred for further evaluation, and endodontic surgery or extraction if the tooth was contraindicated for endodontic surgery as in case of vertical root fracture after REPs [21]. Clinically, teeth with slight sensitivity to percussion or palpation with the absence of pain, swelling, sinus or fistulous tract formation, or abnormal tooth mobility were reported successful.

Pain perception in response to the TPT was reported as “positive” or “negative “according to the presence or absence of pain. Regarding EPT readings, based on the manufacturer guideline, pain or tingling sensation at the following if the reading values: 1) “0 to 39” indicated tooth vitality, 2) “40” to “79” indicated partial tooth non-vitality, and 3) “80” indicated non-vital tooth.

### Standardization and calibration

All clinical procedures were performed by an expert operator in the REPs (9 years of clinical experience). The trial’s outcomes were assessed at each follow-up interval by two independent experts (PhDs in endodontics and paediatric dentistry). The inter-observer reliability using the coefficient of Cohen’s kappa (k) was used. For clinical evaluation, k was 1.00, while for PAI scores and pulp sensibility tests, the inter-observer reliability scores were 0.92 and 0.96, respectively.

### Statistical analysis

The distribution of demographic data (gender and tooth type), clinical and radiographic outcomes, and TPT records in both groups was compared using the chi-square test and the Fisher exact test. The participants’ mean ages in the two groups were tested using the sample *t-test*. The Kolmogorov–Smirnov and Shapiro–Wilk tests were firstly used to assess the fulfilment of PAI scores and EPT records to the assumption of normality. The PAI values of both groups were tested using the non-parametric Mann–Whitney U test.

The main impact of time on the PAI values and the interaction between time and REPs technique were assessed using the general linear model (GLM).

All statistical analysis test were two-sided and performed using the IBM SPSS Statistics 27 software (IBM Corp., Armonk NY, USA). Statistical significant difference was considered when the *p*-value was less than 5% (an alpha level of significant was <0.05).

## Results

Of 48 adolescents with 54 teeth examined for the eligibility of the enrolment in the current trial, forty-four patients with 48 maxillary central incisors fulfilled the inclusion requirements. Four patients with 6 teeth were excluded because of the presence of a broken file that couldn’t be retrieved from the root canal (one tooth), unrestorable crowns that needed post insertion (4 teeth), and the parent of the last adolescent refused to participate in the trial (one tooth).

Data in Table 1. revealed demographic data of the participants, clinical and radiographic outcomes of the BC and PRF groups were not statistically significant over the whole follow-up intervals (*P* > 0.05). After 3 months, the clinical and radiographic outcomes were favourable in both groups with a success rate of 100%. After 6 months, only one tooth (4%) in the BC group showed a fistulous formation with an increase in the dimensions of the periapical lesion. After 12 months, two patients with two teeth in each group failed to attend the follow-up appointment (*n* = 22 teeth/group). An additional participant with one tooth couldn’t be assessed after 24 months (*n* = 21 teeth/group). After 12 and 24 months, the clinical and radiographic success rates of the BC group were 96% and 95%, respectively. While no clinical or radiographic failures were detected in the PRF group at all scheduled follow-up intervals with success rates of 100%.

**Table 1:** Participants’ baseline criteria, clinical and radiographic outcomes of the two regenerative techniques, blood clot (BC) and platelet-rich fibrin (PRF)

Variables	BC	PRF	*P*-value
Gender			
Male	11 (46)	12 (50)	0.77
Female	13 (54)	12 (50)	
Age (years)			
Mean ± SD	15.25 ± 1.33	15.04 ± 1.37	0.60
Tooth type			
Right central incisor	9 (38)	8 (33)	0.76
Left central incisor	15 (62)	16 (67)	
Clinical outcomes (success)			
At 3 months	24/24 (100)	24/24 (100)	1.00
At 6 months	23/24 (96)	24/24 (100)	0.50
At 12 months	21/22 (96)	22/22 (100)	0.50
At 24 months	20/21 (95)	21/21 (100)	0.50
Radiographic outcomes (reduction in the periapical radiolucency)			
At 3 months	24/24 (100)	24/24 (100)	1.00
At 6 months	23/24 (96)	24/24 (100)	0.50
At 12 months	21/22 (96)	22/22 (100)	0.50
At 24 months	20/21 (95)	21/21 (100)	0.50

Chi-square test for gender and tooth type; Independent t-test for age, Fisher exact test for clinical and radiographic outcomes. *P*-value set at ≤0.05

**Fig. 3:**
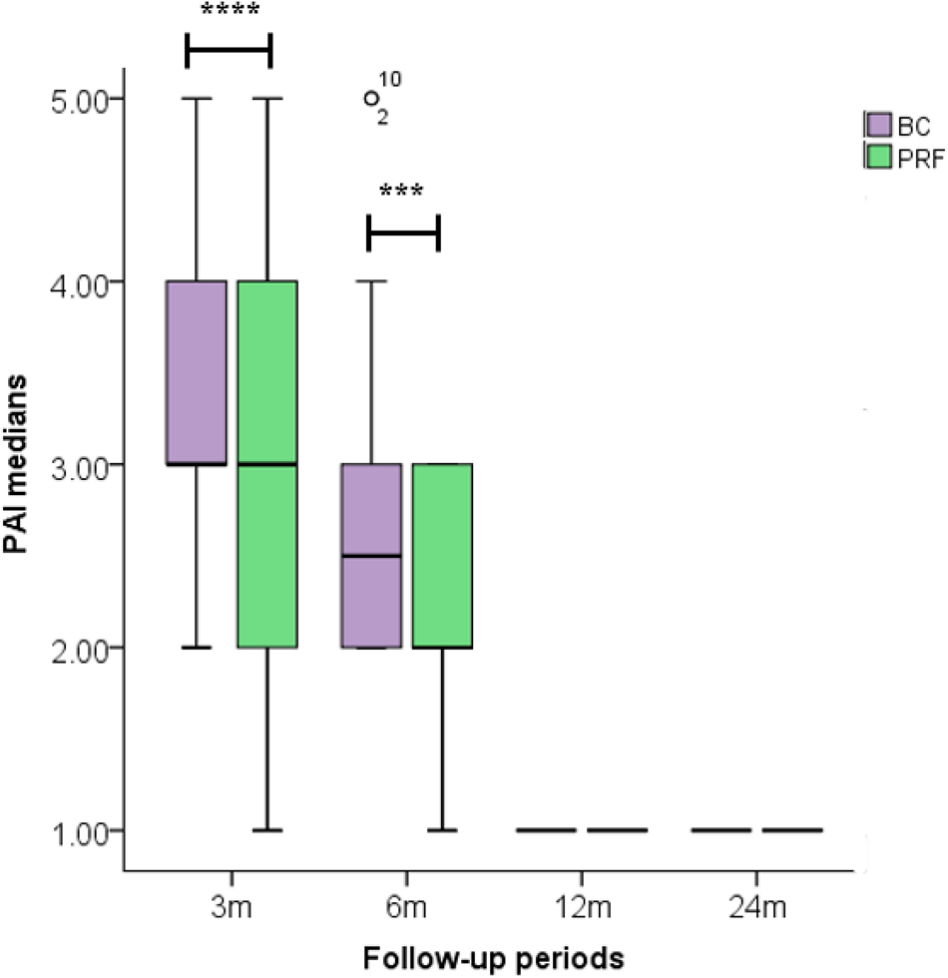
Periapical index (PAI) median scores for retreated maxillary central incisors after retreatment with BC formation (BC) and platelet-rich fibrin (PRF) at different follow-up intervals. The difference between the BC and PRF groups was tested using Mann–Whitney U test, which was statistically significant after 3 and 6 months. While the PAI medians were similar in both groups after 12 and 24 months. *P ≤ 0.05, ** *P* ≤ 0.01, ***P ≤ 0.001, **** *P* ≤ 0.0001.

**Fig. 4:**
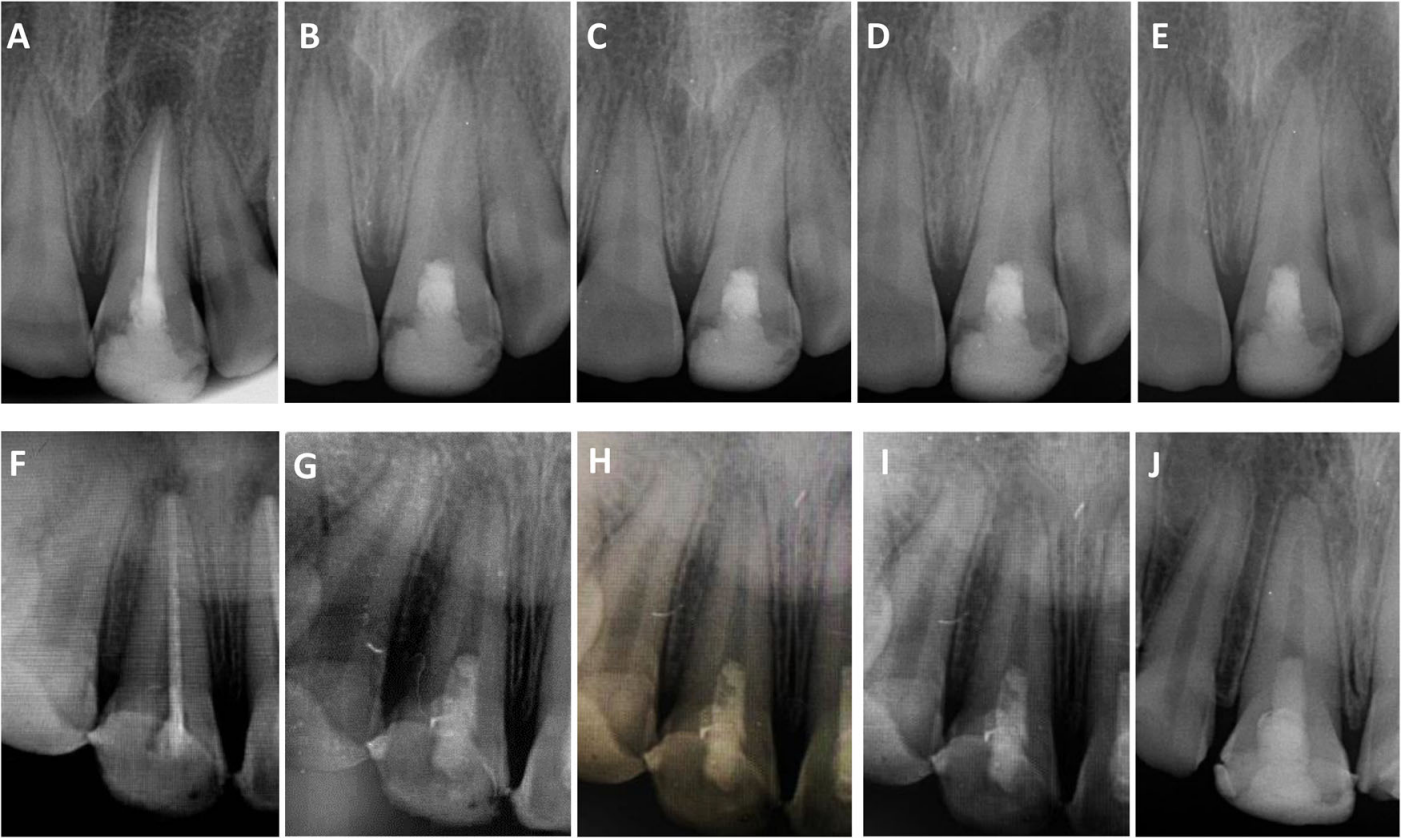
Periapical radiographs (PAs) showed the scores of periapical index (PAI) at different follow-up intervals. The first case (A$-$E) was for a 15-year-old female with maxillary left central incisor retreated with platelet-rich fibrin (PRF). The PAI scores at: **A** baseline, was 4, **B** after 3 months was 2, **C** after 6 months was 1, **D** after 12 months was 1, and **E** after 24 months was 1. The second case (F$-$J) was for a 14-year-old male with maxillary left central incisor retreated with induced blood clot (BC) formation. The PAI scores at: **F** baseline, was 4, **G** after 3 months was 3, **H** after 6 months was 2, **I** after 12 months was 1, and **J** after 24 months was 1.

The PAI medians for the matched groups at the baseline was 3.5. As illustrated in Fig. 3, the PAI medians were significantly lower in the PRF group after 3 months (*P* < 0.0001) and 6 months (*P* = 0.002), indicating faster resolution of the periapical lesions in the platelet-concentrate scaffold group. While after 12 and 24 months, the PAI median were similar in the two REPs. PAs in Fig. 4 monitored the healing progress over different follow-up periods of two retreated maxillary central incisors with PRF and induced BC formation.

**Fig. 5:**
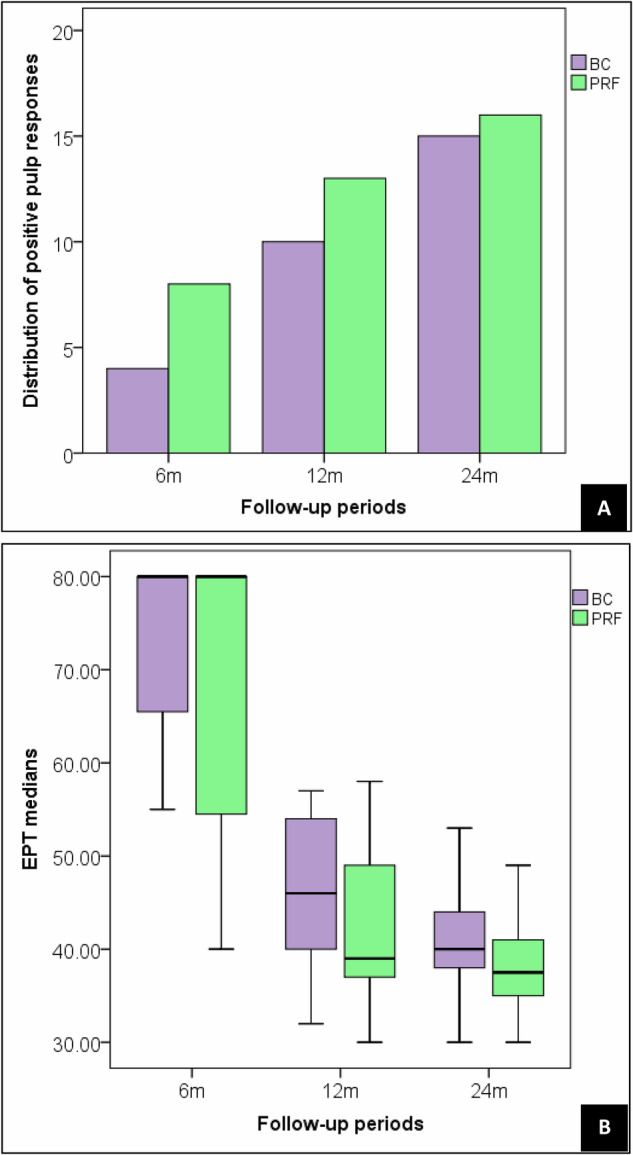
Response to pulp sensibility tests at follow-up intervals. **A** Thermal pulp test (TPT) distribution, **B** Electric pulp test (EPT) medians.

Regarding pulp sensibility, retreated maxillary incisors with the two REP techniques showed no response to TPT or EPT after 3 months. Over the follow-up periods, the number of teeth that positively responded to the thermal stimuli was not statistically significant. After 6 months, four teeth (17%) and eight teeth (34%) were positively responded to the cold stimuli. After 12 months, positive pulp responses to TPT were detected in 42% (n = 10/22) and (59% n = 13/22) teeth in the BC and PRF groups, respectively. After 24 months, positive pulp responses were increased to 71% (n = 15/21) in the BC group and 73% (n = 16/22) in the PRF group. The EPT mean values after 12 and 24 months in the BC group were 46.33 ± 7.70 (median = 46) and 40.86 ± 6.60 (median = 40), respectively. For the PRF group, the mean of EPT scores after 12 months was 42.23 ± 8.15 (median = 39), and after 24 months it was 37.9 ± 15.22 (median = 39.5). The difference between the positive responses to TPT and EPT after 12 and 24 months was not statistically significant as represented in Fig. 5.

The main effect of time on the PAI scores. Time had a statistically significant impact on the PAI scores over the follow-up periods (*P* < 0.0001) with an effect size of 0.298. While the interaction effect of time with the REPs technique was not statistically significant, indicating that time affected the PAI scores similarly for both techniques (*P* = 0.126) with an effect size of 0.059.

## Discussion

Recently there has been a gradual increase in considering the REPs in prospective clinical experiments for treatment of mature permanent teeth. However, the evidence firmly supporting regeneration/revitalization for apical periodontitis in immature or mature permanent teeth remains of low-quality [12].

In the present trial, REPs were considered for the retreatment of mature teeth even though conventional root canal retreatment is a viable option for the following reasons: 1) REPs are generally less expensive than conventional retreatment because they often require fewer appointments and less complex instrumentation. Conventional retreatment involves removing existing root canal fillings, re-instrumenting the canals, and re-obturating, which can be time-consuming and costly. In contrast, REPs focus on disinfection and revitalization of the pulp-dentin complex, which may reduce the need for additional procedures such as apical surgery or extraction followed by implant placement.2) REPs simplify the process by focusing on disinfection and promoting natural tissue regeneration rather than mechanical debridement and obturation. Additionally, REPs reduce the need for complex instrumentation and minimises the risk of procedural errors [22]. 3) Improve the patient’s outcomes because REPs aim to restore the natural biology of the tooth by promoting the regeneration of pulp-like tissue, which can enhance tooth vitality and function. This contrasts with conventional retreatment, which often results in a non-vital tooth that is more prone to fracture and failure over time. REPs also preserve the natural tooth structure, which is beneficial for long-term oral health [23]. 4) REPs are less invasive and offer the potential for natural tissue regeneration.

However, the use of REPs in the retreatment of mature permanent teeth can be complicated by several factors. The complex root canal anatomy may hinder complete disinfection, increasing the risk of persistent infection and failure of the regenerative procedure. Additionally, cases with insufficient coronal structure or advanced periodontal disease may not be suitable for regenerative endodontics [24]. Nevertheless, ongoing research may expand the indications for REPs in the future.

Although the treatment of mature necrotic permanent teeth with apical periodontitis using REPs is still in its early stages, the limited available studies have shown promising outcomes. This encouraged us to explore the potential benefits of REPs in the retreatment of mature maxillary central incisors with periapical lesions. Therefore, this study hypothesized that there would be no clinical or radiographic difference in periapical lesion healing following the retreatment of mature maxillary central incisors with two REP techniques —revascularization with blood clot (BC) formation versus platelet-rich fibrin (PRF)—over a 24-month period. The other outcome was to assess the pulp responses after thermal and electrical conduction tests.

In the present study, although the difference between the BC and PRF groups was not significant, PRF may offer certain advantages, particularly in inducing bleeding, which was not always possible from the periapical region [25]. One disadvantage of BC is its relatively fragile structure, which may prevent it from completely filling the RC, potentially compromising the coronal seal [26]. Additionally, a systematic review reported deposition of hard tissue in all studies that depended on PRP and PRF scaffolds, while the mineralized tissues were reported in 80% of studies that considered BC in regeneration [27]. However, the need for specialized equipment to collect the venous blood and prepare the PRF was probably the main problem. Moreover, the PRF technique may increase treatment complexity, time and cost, and patient cooperation is necessary.

The impact of age on the regenerative capacity still being controversial. Arslan et al. [13] reported that the age of participants aged 10$-$35 years had no influence on the outcomes of REPs. In contrast, a report of expert consensus on REPs emphasized the significance of age in achieving successful regenerative outcomes [6]. Therefore, this study focused on adolescents to standardize anatomical and physiological factors, such as tooth morphology, root number (only single-rooted maxillary central incisors were included), root length and thickness, and apical foramen size and position. These factors may influence the influx of stem cells and the response to pulp sensibility tests. Additionally, the age limitation aimed to account for the age-related regenerative capacity of stem cells [28].

The biological influence of a patient’s age can be attributed to the reduced potential for revascularization, which is associated with degenerative changes in blood vessels. These changes coincide with continuous dentin apposition and an increased incidence of calcifications [29]. Additionally, the regenerative capacity of multipotent stem cells declines with age [30]. This decline is likely due to the reduced differentiation potential of mesenchymal stem cells (MSCs) over time rather than a decrease in the number of MSCs migrating into the root canal system, as this population remains relatively stable with aging [31].

The diameter of apical foramen is another factor that may influence the REPs outcome. Nevertheless, the ideal apical foramen size has not yet been established. According to the findings of Fang et al. [32], mature teeth with apical foramen diameters ranging from 0.5 to 1.0 mm had the most favourable outcomes with a clinical success rate of 95.65%. This result is consistent with the findings of the present study. Some reports indicate that REPs have higher clinical and radiographic success rates in younger patients. For example, a study found that nine out of ten maxillary incisors with apical foramen diameters between 0.5 and 1.0 mm in adolescents aged 14–18 showed clinical and radiographic success after one year of follow-up. Additionally, findings from an animal model study suggest that a minimum apical terminus diameter of 0.4 mm is required for successful PEPs [33].

The PAI method was used to track healing status by assessing changes in periapical radiolucency. PAI is a widely used, standardized, and reproducible quantitative method with high inter- and intra-examiner reliability [34, 35]. To enhance reproducibility and diagnostic accuracy, a customized radiographic stent was used for each patient, ensuring consistent angulation and minimizing variations in imaging geometry. Additionally, two calibrated examiners independently scored the radiographic images.

Despite the superiority of three-dimensional imaging techniques such as cone-beam computed tomography (CBCT), this study relied on conventional two-dimensional PAs. CBCT is not recommended for the routine radiographic diagnosis of teeth with periapical periodontitis unless there is a discrepancy between conventional radiographic findings and clinical signs and symptoms [36, 37]. Additionally, ethical concerns regarding CBCT use in children and adolescents must be considered. Growing children are significantly more sensitive to ionizing radiation than adults [38]. Therefore, the potential benefits must be carefully weighed against possible risks, and each case should be evaluated individually to justify the use of CBCT [39].

The current trial outcomes demonstrated the effectiveness of REPs in the retreatment of mature permanent incisors, with no clinically or radiographically significant differences between BC formation and PRF techniques. However, the PAI scores after retreatment with PRF significantly decreased compared to those treated with BC formation at both 3 and 6 months. This suggests that healing occurred more rapidly in teeth retreated with the PRF clot.

This accelerated healing may be attributed to the combination of intentional bleeding induced from the periapical region and the PRF scaffold. This combination likely increases the presence of stem cells and enhances the sustained release of growth factors and cytokines over time. The PRF scaffold, which contains highly concentrated platelets, provides a rich source of bioactive molecules and mesenchymal stem cells (MSCs), promoting greater stem cell proliferation and improving overall healing [40].

Given the limited availability of prior data addressing this topic, a direct comparison of the outcomes of the present trial with previous findings is challenging. Only one randomized trial has investigated the impact of regenerative endodontic procedures (REPs) using blood clot (BC) revascularization on the retreatment of mature permanent incisors with apical periodontitis in adolescents [15]. In that study, involving thirty-three permanent incisors, the clinical success rate at 6 and 12 months was 93.9%, which aligns closely with the results observed in the current study at similar follow-up intervals. However, after 6 months, the PAI median in the previous trial decreased significantly to 1.5, whereas in the present study, the PAI median decreased to 2. This discrepancy may be attributed to differences in baseline PAI medians, which were higher in the present investigation. Additionally, variations in sample size could also contribute to the differences in PAI medians between the two studies.

The findings from the current study confirmed that the difference in clinical success was not significant. This aligns with a recently published study involving 51 mature, permanent, single-rooted teeth with chronic apical periodontitis (PAI > 3). In that study, the teeth were treated using three different approaches: BC formation, standard-PRF, and advanced-PRF, with 17 teeth in each group [41]. The success rates of S-PRF and A-PRF were similar (88.2%), while the BC group had a success rate of 82.4% after eighteen months of follow-up. Another trial, using a similar methodology and age group, reported a clinical success rate of 93.9% for maxillary incisors treated with BC formation, compared to 97% for teeth that underwent conventional retreatment after twelve months of follow-up [15]. Similarly, in cases of immature teeth, most failures were identified within one year after REPs, with the primary cause being persistent periapical infection, accounting for 79% of failures [25].

The long-term outcomes observed in this study are consistent with the findings of a few prior randomized clinical trials that have investigated REPs in the primary root canal treatment of mature teeth with apical periodontitis. For instance, a study by Jha et al. [42], examined 15 mature permanent teeth in children aged 9–15 years treated with BC formation. The study demonstrated a consistent decline in mean PAI values over 18 months period with regular follow-ups. The preoperative mean PAI value of 3.60 decreased to 1.60, 1.40, and 1.10 at subsequent follow-ups, respectively, with an overall success rate of 100%.

Other trials have investigated REPs in older patient groups. One trial reported a clinical and radiographic success rate of 92.3% in patients treated with the BC approach [13]. Another study compared radiographic healing outcomes between two REP techniques—BC and platelet-PRF—in 20 teeth (10 per group). After 12 months, significant healing of periapical lesions was observed, with median PAI scores decreasing from 3.5 and 4 to 2 and 1 in the BC and PRF groups, respectively [5]. Furthermore, a randomized clinical trial assessed the efficacy of MTA and Biodentine in the revascularization of mature permanent incisors with apical lesions. Over an 18-month follow-up, complete healing was observed in 67.7% of treated teeth, while the remaining 32.3% showed progressive healing. These findings highlight the therapeutic potential of REPs in managing mature permanent teeth with periapical lesions [43].

In line with current findings, a study evaluated forty-five mature and immature single-rooted permanent teeth with apical periodontitis using conventional periapical radiographs and CBCT after treatment with three different regenerative endodontic procedures (REPs): blood clot (BC), platelet-rich fibrin (PRF), and platelet-rich plasma (PRP). CBCT analysis revealed that teeth treated with PRF showed the highest median reduction in periapical lesion size (69.57%), followed by BC (44.02%) and PRP (23.08%). However, the differences were not statistically significant [44]. A recent randomized clinical trial used CBCT to evaluate the healing of periapical lesions in eighteen necrotic maxillary permanent incisors. The results showed no significant difference between teeth treated with PRF and those treated with concentrated growth factor (CGF) over a follow-up period of 6 and 12 months [45].

Histological analysis of regenerated tissues following REPs in mature teeth has revealed the formation of mineralized tissue resembling bone (osseous-like) and cementum (cementum-like). These tissues are embedded within dense, highly vascularized, fibrous periodontal-like tissue, but without the induction of reparative dentin due to the absence of odontoblasts [46]. A previous study confirmed the histological similarities between the regenerated connective tissue—containing osseous-like tissue, cementum-like tissue, and vascular components—after REPs in both immature and mature permanent teeth [47].

Regarding the response to the pulp sensibility tests, combining TPT and EPT in the current study was considered. A previous literature review advocated this combination because the outcomes of one test can corroborate the results obtained from the other test, thereby enhancing the reliability and validity of the findings through cross-verification [48].

Previous studies have reported varied pulp sensibility outcomes. A prior investigation on the retreatment of mature incisors with apical periodontitis found positive pulp responses in 54.54% of cases after one year, following the formation of a biological barrier (BC) [15]. This success rate was higher than that observed in the present study, which utilized a revascularization approach. The discrepancy may be attributed to the reliance on EPT alone, whereas the current study employed a combined TPT and EPT methodology.

In the present study, positive pulp responses were observed in 59% of PRF-based retreated mature teeth after one year, increasing to 73% after two years. These results align with previous studies, which reported positive pulp responses in 50% of PRF-treated teeth [5, 13]. Two other studies reported success rates of 60% [10], and over 60% [49] for mature teeth treated with BC formation, which is higher than the 42% observed in the present study after 12 months. This variation may be due to differences in the preoperative status of the included teeth (i.e., presence or absence of periapical radiolucency). However, after 24 months, comparable positive responses were recorded.

Positive responses to pulp sensibility tests in both the blood clot and PRF groups are encouraging, suggesting that both methods may effectively preserve or restore pulp vitality in the short term. However, long-term success depends on ongoing monitoring, the absence of inflammation or infection, and the pulp’s ability to heal and maintain its function over time. Further research, including long-term clinical studies, would be beneficial to compare the effectiveness of these methods in ensuring the lasting success of retreatment procedures.

Although the difference in pulp sensibility records between the BC and PRF techniques was not significant, the faster healing and the higher number of teeth responding positively to pulp sensibility tests in the first year after retreatment with PRF could be due to the abundant and sustained release of growth factors—such as TGF-β, PDGF, insulin-like growth factor 1, and bFGF—for 7–28 days, which mediate new vascular and nerve fiber formation [10, 50]. Additionally, the presence of leukocytes, cytokines, and lymphocytes in PRF creates a balanced and self-regulating system that effectively manages inflammation and infection while enhancing the healing potential of the PRF clot [10].

The findings of the present study confirm the success of REPs even in the presence of infection and inflammation in the pulp and periapical tissues. This might be due to the presence of residual vital pulp tissue around the root apex [6]. Generally, the mature teeth possess a lesser number of stem cells than those available in the immature teeth [51]. Upon evoking apical bleeding, periodontal ligament stem cells (PDLSCs), bone marrow mesenchymal stem cells (BMSCs), and residual dental pulp stem cells (DPSCs) around the root apex of mature necrotic teeth migrate into the canal space, organize on the three-dimensional matrix, and contribute to the repair process [51, 52].

Despite the relatively long follow-up period in the current study, this trial may offer a new perspective on the retreatment of mature teeth with apical periodontitis, rather than relying exclusively on conventional root canal retreatment. However, certain limitations should be acknowledged, such as the reliance on two-dimensional radiographs to assess periapical healing, which is less sensitive than three-dimensional imaging [53]. Nevertheless, some reports have demonstrated consistency between two-dimensional and three-dimensional imaging techniques, such as CBCT [7, 54]. Further clinical trials incorporating CBCT for a subset of cases could provide a more detailed assessment of healing, particularly in complex cases.

To expand the benefits, studies should include other age groups, varying apical foramen sizes, and different tooth types. Another limitation is the relatively small number of treated teeth, which were confined solely to maxillary mature permanent teeth. This may restrict the generalizability of the findings. Therefore, larger cohort randomized clinical trials with at least five years of follow-up are needed to assess the durability of outcomes, the potential for late failures, and to provide more robust evidence. Additionally, histological studies are essential to determine the nature of newly regenerated tissues after the retreatment of mature teeth and to validate the regenerative potential of REPs in mature teeth.

Finally, relying solely on sensibility tests presents challenges, as a positive response does not necessarily indicate true pulp regeneration. Alternative methods, such as laser Doppler flowmetry or pulse oximetry, may offer more accurate and reliable data regarding the actual pulp status of retreated mature permanent teeth.

While REPs have shown promise in treating immature teeth with necrotic pulps, their application in mature teeth presents several significant challenges. One of the primary limitations is the difficulty of achieving adequate disinfection in mature teeth with complex root canal systems. These teeth often have narrower root canals, denser dentin, and intricate anatomical variations, such as lateral canals and isthmuses, which can harbour biofilm and hinder effective disinfection [1]. Another major challenge is the potential for incomplete regeneration of the pulp-dentin complex. Unlike immature teeth, mature teeth have a limited blood supply and a less favourable microenvironment for stem cell recruitment and differentiation [23].

The regenerative potential of REPs in mature teeth is further limited by the reduced number of resident stem cells and the diminished capacity for angiogenesis and neurogenesis. Consequently, the outcomes of REPs in mature teeth are often less predictable, with a higher likelihood of fibrous or osseous tissue formation rather than true pulp regeneration. Additionally, clinical protocols for REPs in mature teeth are not yet standardized, leading to variability in treatment outcomes. Factors such as the choice of irrigants, intracanal medicaments, and scaffold materials can significantly influence the procedure’s success [55].

Although conventional root canal retreatment remains the gold standard for retreating mature permanent teeth, the promising outcomes of REPs in previous clinical trials encouraged us to explore and compare alternative REP techniques (BC vs. PRF). This paradigm shift in treatment approach also necessitates a shift in the assessment of clinical outcomes. Therefore, this trial aimed to compare two corresponding REP approaches. Further research comparing different REP techniques with conventional root canal retreatment is needed to optimize treatment protocols and develop strategies to enhance the regenerative potential of REPs in mature teeth.

Additionally, multicenter studies with larger sample sizes and a follow-up period of at least five years, utilizing advanced imaging techniques and histological investigations to validate regenerative outcomes, are mandatory.

## Conclusions

Both techniques show promise with comparable results in terms of clinical and radiographic outcomes. The healing of periapical radiolucency was significantly faster in the PRF-retreated teeth compared to those retreated with BC approach after 3 and 6 months. However, after 12 and 24 months, the PAI scores were similar. The number of positive response to the pulp sensibility test was approximately similar in the two REPs groups after 24 months of the follow-up.

## Data Availability

All data generated or analysed are include in the article.
